# Reusability Report: evaluating the performance of a meta-learning foundation model on predicting the antibacterial activity of natural products

**DOI:** 10.21203/rs.3.rs-6932613/v1

**Published:** 2025-08-12

**Authors:** Allison Walker, Caitlin Butt

**Affiliations:** Vanderbilt University; Vanderbilt University

## Abstract

Deep learning foundation models are becoming increasingly popular for their use in bioactivity prediction. Recently, Feng et al. (Nature Machine Intelligence 2024), developed ActFound, a bioactive foundation model that jointly uses pairwise learning and meta-learning. By utilizing these techniques, the model is capable of being fine-tuned to a more specific bioactivity task with only a small amount of new data. To investigate the generalizability of the model, we looked to fine-tune the foundation model on an antibacterial natural products (NPs) dataset. Large, labeled NPs datasets, which are needed to train traditional deep learning methods, are scarce. Therefore, the bioactivity prediction of NPs is an ideal task for foundation models. We studied the performance of ActFound on the NPs dataset using a range of few-shot settings. Additionally, we compared ActFound’s performance with those of other state-of-the-art models in the field. We found ActFound was unable to reach the same level of accuracy on the antibacterial NPs dataset as it did on other cross-domain tasks reported in the original publication. However, ActFound displayed comparable or better performance compared to the other models studied, especially at the low-shot settings. Our results establish ActFound as a useful foundation model for the bioactivity prediction of tasks with limited data. Especially for datasets that contain the bioactivities of similar compounds.

The bioactivity of compounds plays a key role in drug discovery. Bioactivity refers to the effect, either beneficial or adverse, a compound has on a biological process^1^. It encompasses the efficacy, potency, and selectivity of a compound and is important in the identification of hits in a drug campaign and subsequent lead optimization^2^. Deep learning (DL) approaches have shown promise in their ability to predict the bioactivity of compounds^3,4,5,6,7^. However, DL models require large, high-quality datasets in order to accurately identify patterns within the data^8^. Many bioactivity tasks do not have an adequate amount of labeled data sufficient for training. One solution is to use foundation models, which are pretrained on large, general datasets. These models serve as a ‘foundation’ which can be fine-tuned for more specific tasks^9^.

Recently, Feng et al. introduced ActFound^10^, a meta-learning foundation model trained to predict the bioactivity of compounds. To accomplish this, ActFound jointly utilizes meta-learning and pairwise learning. Meta-learning, or “learning to learn,” is a commonly used algorithm to develop foundation models^11,12^. Meta-learning models are trained on a variety of tasks with the intention of creating a model that can quickly adapt to new tasks from only a small amount of new data^13^. By using meta-learning, Feng et al. were able to pre-train their model on a wide range of diverse assays, leveraging the information to develop a general foundation model capable of few-shot learning. Pairwise learning was used to address the incompatibility of information within training assays differing in metrics, units, and value ranges. Instead of directly predicting the bioactivity of compounds, pairwise learning allows ActFound to predict the difference in bioactivity between two compounds within the same assay^14^.

During the fine-tuning stage, ActFound utilized the algorithm *k*-nearest neighbors MAML (kNN-MAML), which identifies assays within the training set that are similar to the fine-tuning assay. Leveraging information from similar assays allows for rapid fine-tuning to a new, unseen assay.

In this reusability report, we looked to study ActFound’s performance on a natural products (NPs) dataset that contains plant-derived compounds with antibacterial activity^15^. NPs are an abundant source of antibiotics, with many approved antibiotics being NPs or NP-derivatives^16^. However, antibacterial NPs have historically been plagued by the dereplication process, where known NPs are repeatedly rediscovered^17^. The lack of new antibiotics being identified has led to a rising interest in DL models for antibacterial prediction^18,19,20,21^. However, there is a lack of large, labeled bioactivity datasets in the NPs field^22^. This makes it an ideal task for a pairwise meta-learning model like ActFound. In this study, we fine-tuned both the ActFound model pre-trained on assays from ChEMBL^23^ as well as the model pre-trained on assays from BindingDB^24^. We investigated the use of the few-shot setting, fine-tuning the models on the NPs dataset in a range of shot-settings between 8 and 128 fine-tuning compounds (Fig. 1). We then compared the performance of the ActFound fine-tuned models with other conventional meta-learning models MAML and ProtoNet as well as transfer learning variants of ActFound and MAML^13,25^.

## Fine-tuning ActFound on a Natural Products Dataset

**Figure 2:**
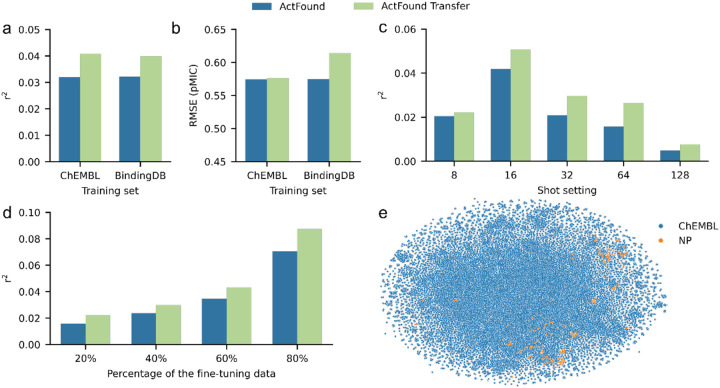
Performance of ActFound on the NPs dataset. **a,b,** Bar plots comparing ActFound and ActFound Transfer’s performance on the NPs dataset in terms of (**a**) r^2^ and (**b**) RMSE. Plot shows the mean value across each shot setting used to fine-tune the ChEMBL pre-trained models and the BindingDB pre-trained models. **c,d,** Bar plots comparing the models’ performances in terms of r^2^ at the (**c**) 8-, 16-, 32-, 64-, and 128-shot setting and when (**d**) 20, 40, 60, 75, and 80% of the assays were used for fine-tuning. (e) t-SNE plot comparing the molecules in the NPs dataset and a random selection of 50% of the ChEMBL training dataset.

To investigate ActFound’s ability to generalize to new domains not explored in the original publication, we fine-tuned the model on an antibacterial NPs dataset. This dataset was curated by Porras et al. in an extensive literature review spanning from 2012 to 2019 and contains the growth inhibitory activity of NPs against a range of bacteria. A t-Distributed Stochastic Neighbor Embedding (t-SNE) comparing the compounds within the NPs dataset and the ChEMBL training data shows overlap, indicating the two datasets likely contain similar compounds (Fig 2e). However, we did not attempt to identify any identical assays between the datasets or exclude molecules potentially seen during training. We acknowledge this could have caused data leakage that inflated the performance of the model pre-trained on ChEMBL assays. Given that this dataset was the result of manual literature curation, it is unlikely that the exact data in this dataset was deposited into ChEMBL. In addition, considering the NPs dataset only contained growth inhibitory assays, there should be no identical assays in the BindingDB training dataset.

**Figure 3:**
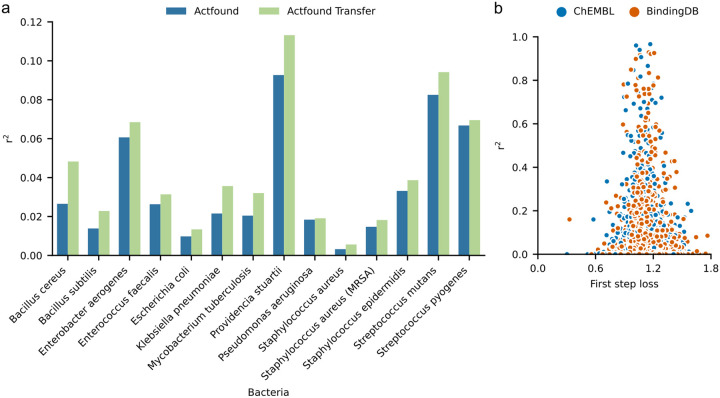
Performance of ActFound on each assay. **a,** Bar plot comparing ActFound and ActFound Transfer’s performance on each assay in the NPs dataset in terms of r^2^. Plot shows the mean value across each shot setting used to fine-tune the ChEMBL pre-trained models and the BindingDB pre-trained models. **b,** Scatter plot comparing the first step loss and r^2^ value for every fine-tuning iteration for each assay for the ChEMBL pre-trained ActFound and the BindingDB pre-trained ActFound.

When fine-tuning, we considered each bacteria strain to be its own assay and assessed the performance of ActFound across a range of shot settings. This included using 8 to 128 fine-tuning compounds as well as using 20 to 80% of the compounds within each assay for fine-tuning. Averaging the r^2^ value across all shot settings for ActFound and ActFound Transfer, a transfer learning variant of ActFound, showed that overall ActFound Transfer had a higher r^2^ value than ActFound on the NPs dataset (Fig 2a). However, ActFound was found to have the lowest RMSE value (Fig 2b). When looking at the performance for each shot setting, ActFound and ActFound Transfer performed the best in the 16-shot setting, with performance dropping as the shot setting increased (Fig 2c). This is in contrast with the original publication, where the performance of ActFound increased with the number of compounds used for fine-tuning. Since providing more compounds for fine-tuning should inherently improve performance, we investigated using a percentage of the assays for fine-tuning instead of supplying a specific shot setting. In this case, ActFound performed as expected, with the r^2^ values increasing as the percentage of compounds used for fine-tuning increased (Fig 2d). We attribute this behavior to the fact that only four assays had enough compounds to be used for fine-tuning in the 64- and 128-shot setting. When looking at the performance on each assay, the four largest assays (*B. subtilis*, *E. coli*, *S. aureus*, and *S. aureus* (MRSA)) were the four assays with the worst model performance (Fig 3a).

ActFound was found to have varying degrees of performance across the 14 assays, ranging from r^2^ values of 0.01 to 0.12. For reference, in the cross-domain setting, the original publication found two kinase inhibitor datasets to have average r^2^ values between 0.15 and 0.25, so the model performs only slightly worse on some of the NP antibacterial datasets and much worse on others. ActFound utilizes pairwise learning and works under the assumption that similar compounds will have similar bioactivities. This is typically advantageous, as many assay data sets are assembled to investigate structure-activity relationships (SAR) thus the compounds will be similar to one another. However, one disadvantage to this approach, and what we believe is causing such a range in performance for the NPs dataset, is that if the assay does not contain similar compounds, the pairwise learning function will cause large errors. In the original publication, Feng et al. removed what they called ‘orphan compounds’ from the assays. These orphan compounds are those which have a Tanimoto similarity less than 0.2 to the other compounds in the assay. When we performed the same procedure, none of the assays had enough compounds left for fine-tuning. This is likely due to how we defined our ‘assays.’ Although Porras et al. provided references in the NPs dataset, there were not enough bioactivities per bacteria in each reference to fine-tune the model. Therefore, we combined compounds from multiple references to treat each bacteria strain as its own assay, which likely led to many dissimilar compounds in each assay. Since removing the orphan compounds left only one assay available for fine-tuning, we decided not to remove them. We acknowledge that this decision likely played a part in how well ActFound was able to perform on the NPs dataset. However, it also reveals an important limitation of the ActFound method. NP datasets will often lack closely related pairs of compounds, as natural products are highly diverse^26^ and congeners of a primary product may be difficult to discover and isolate without the use of specialized techniques^27^. This limitation may also pose a problem for high throughput screening assay datasets that use compound libraries assembled for compound diversity over preliminary SAR, as is sometimes the case^28^.

The original paper found a correlation between a small loss value for the first optimization and a large r^2^ value. This was identified as a way to determine how well ActFound will perform on the fine-tuning assay since assays with small loss values will likely result in high r^2^ values. However, we did not find this correlation to hold on the NPs dataset. Although most of the assays had larger loss values, even the assays with small loss values had small r^2^ values (Fig 3b).

## Evaluation against other state-of-the-art models

**Figure 4:**
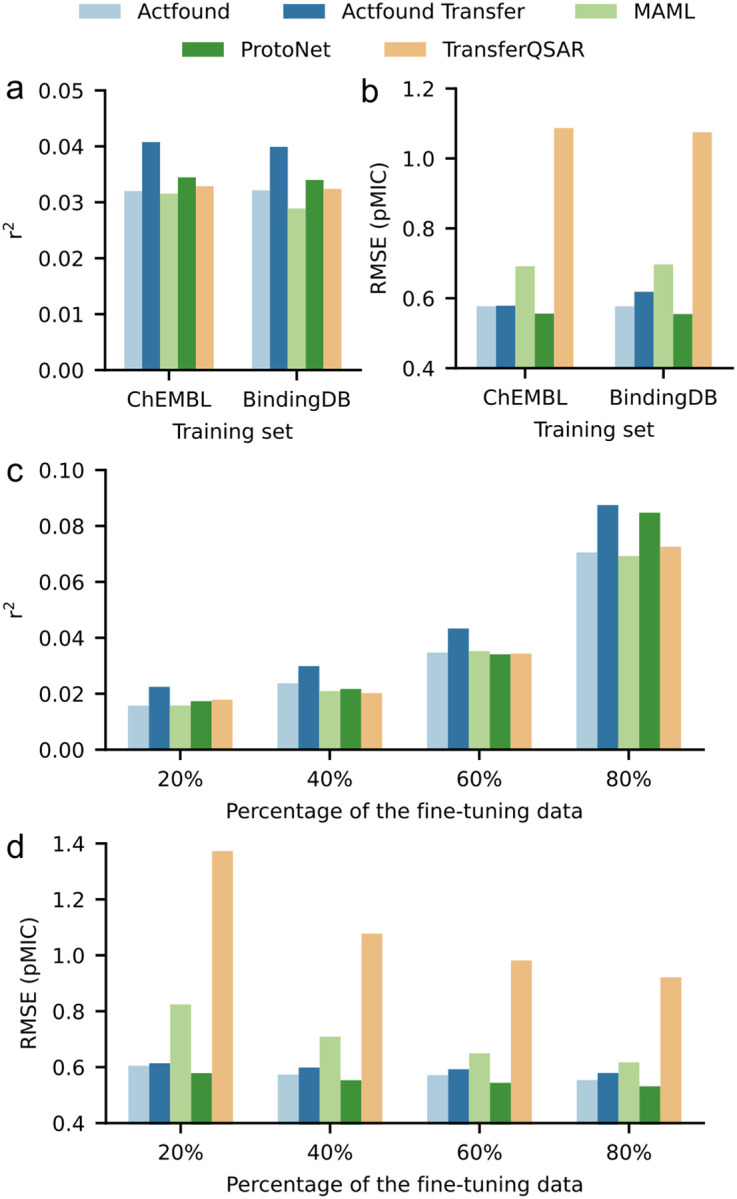
Performance of conventional models on the NPs dataset. **a,b,** Bar plots comparing each model’s performance on the NPs dataset in terms of (**a**) r^2^ and (**b**) RMSE. Plot shows the mean value across each shot setting used to fine-tune the ChEMBL pre-trained models and the BindingDB pre-trained models. **c,d,** Bar plots comparing the models’ performances when 20, 40, 60, 75, and 80% of the assays were used for fine-tuning in terms of (**c**) r^2^ and (**d**) RMSE.

In addition to ActFound and ActFound Transfer, we also used the NPs dataset to fine-tune three other conventional models: MAML, ProtoNet, and TransferQSAR. MAML and ProtoNet are meta-learning models, while TransferQSAR is a transfer learning variant of MAML. However, none of the models incorporate pairwise learning to learn the relative bioactivities as ActFound does. ActFound Transfer outperformed all other models with both the ChEMBL and BindingDB versions (Fig 4a,b). Even though we found ActFound to perform worse on the NPs dataset compared to the results in the original publication, ActFound and ActFound Transfer tended to have a larger r^2^ value than the other three models when fewer compounds were used for fine-tuning (Fig 4c,d). This is indicative of ActFound’s ability to quickly adapt to new assays with only a small number of fine-tuning compounds. However, the meta-learning variant of ActFound did not perform better than the other three models when only 20% of the assays were used for fine-tuning. At this setting, the median number of compounds used for fine-tuning was 9, which is a smaller shot setting than was studied in the original paper. Although we identified disadvantages to using pairwise learning with our dataset, these results also indicate the effectiveness of utilizing pairwise learning to learn the relative bioactivity values. When the number of fine-tuning compounds is increased, both variants of ActFound perform on par with the other models, a trend that was also identified by Feng et al..

## Discussion and Conclusion

To investigate ActFound’s reusability, we fine-tuned the model on an antibacterial NPs dataset, studying its performance on a range of shot settings. With the availability of Feng et al.’s Google Colab, we found ActFound to be easy to use and fine-tune on our dataset. In contrast to the results of the original publication, ActFound Transfer was found to perform better than the meta-learning variant of ActFound. We also found both variants of ActFound to have diminished performances compared to the cross-domain setting in the original paper. This was likely due to the incompatibility of ActFound to the NPs dataset as the assays in this dataset contained dissimilar compounds. This meant the pairwise learning function of ActFound was not able to be fully advantageous for our dataset. Another possible explanation for the relatively low accuracy, is that NPs have different chemical properties than synthetic compounds, which likely make up most of the training data. However, given that the t-SNE analysis shows that the chemical space of the NP dataset overlaps with the training set, we believe that the lack of suitable pairs of compounds for pairwise learning contributes more to lowering the accuracy. Despite the poorer performance on our chosen dataset, we found both variants of ActFound to perform better than other state-of-the-art models at the lower shot settings. Therefore, we believe ActFound to be a very useful framework for those who do not have enough labeled data to train a task-specific deep learning model. Especially for those whose datasets consist of structure-activity relationship studies as these datasets will contain the bioactivities of similar compounds which will increase the capabilities of the pairwise learning function.

## Methods

### Dataset Preparation

The NPs dataset used in this reusability report was obtained from the Porras et al. review paper^15^. To prepare the dataset for fine-tuning, we followed a similar pipeline as Feng et al. did when evaluating ActFound on two kinase inhibitor datasets, KIBA^29^ and Davis^30^. In this cross-domain setting, they considered each kinase to be its own assay. Similarly, we considered each bacteria strain as a separate assay. The NPs dataset contains 1439 growth inhibitory values of 472 unique compounds against 115 bacteria strains. We considered resistant strains and subspecies as separate assays from the original strain. Assays with fewer than 20 compounds were removed. If an assay contained duplicate compounds, the growth inhibitory values were averaged across the duplicated compounds. Additionally, we only considered compounds with MIC values and ug/mL units. This left us with 14 assays with an average of 64 compounds per assay.

### Model Fine-tuning

All of the foundation models (ActFound, ActFound Transfer, MAML, ProtoNet, and TransferQSAR) trained by Feng et al. were obtained from their Figshare at https://figshare.com/articles/dataset/ActFound_data/24452680. We fine-tuned the models using the public code of ActFound from its GitHub repository at https://github.com/BFeng14/ActFound.git. During the fine-tuning process, the hyperparameters and architecture for each model were the same as used by Feng et al. The input for the models were 2,048-dimensional Morgan fingerprints, which were computed using RDKit^31^, and the negative log of the MIC values p(MIC) = −log_10_(MIC).

Each model was fine-tuned using the 8-, 16-, 32-, 64-, and 128-shot settings. Additionally, each model was fine-tuned on different proportions of the data. During this fine-tuning stage, we used 20%, 40%, 60%, and 80% of the assay data for fine-tuning. Each assay was randomly split 40 times into the fine-tuning and testing sets and the models were fine-tuned with each random split. The results of the models are an average across each iteration.

### t-Distributed Stochastic Neighbor Embedding (t-SNE)

To study the similarities between the compounds in the ChEMBL training set and the NPs dataset, we performed a t-SNE analysis using scikit-learn’s t-Distributed Stochastic Neighbor Embedding^32^. A t-SNE reduces the dimensionality of the Morgan fingerprints from 2,048 to 2. We used the default values for each parameter except the distance metric which we set to ‘jaccard’. Since the Jaccard distance, or the Tanimoto distance, is equal to 1 – Tanimoto similarity, the distance between points is directly related to the similarity between compounds. The ChEMBL training set is large (1.4 million datapoints) and running a t-SNE on the entire dataset would be computationally expensive. Therefore, to speed up the computation, we opted to randomly select 50% of the training set to perform the t-SNE.

## Figures and Tables

**Figure 1:**
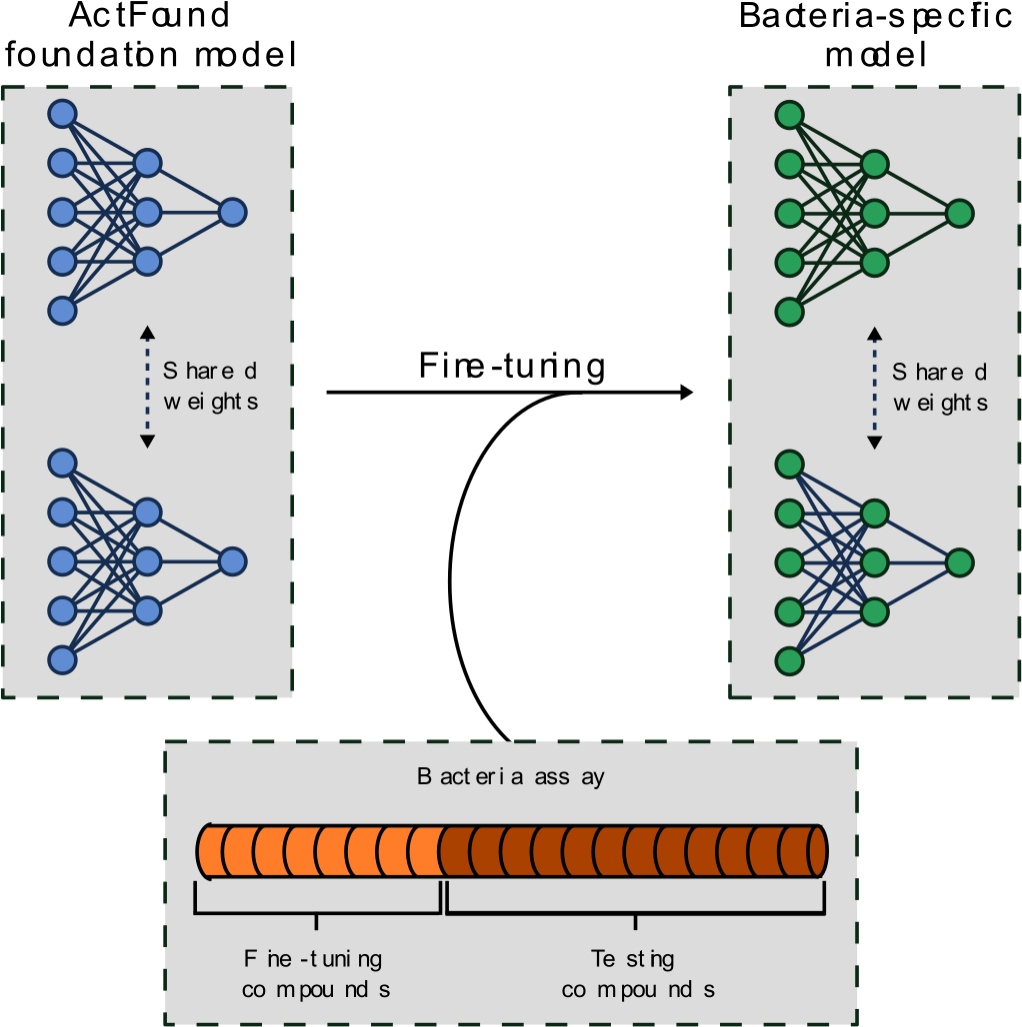
Overview of the fine-tuning procedure. Growth inhibitory assays were used to fine-tune ActFound and create bacteria-specific models.

## Data Availability

The data used in this report are available in ref.^15^.

## Code availability

The ActFound code is available at https://github.com/BFeng14/ActFound.git. All of the foundation model checkpoints are available at https://figshare.com/articles/dataset/ActFound_data/24452680.
